# Newcomer Status as a Protective Factor among Hispanic Migrant Workers for HIV Risk

**DOI:** 10.3389/fpubh.2014.00216

**Published:** 2014-11-07

**Authors:** H. Virginia McCoy, Nancy Shehadeh, Muni Rubens, Christi M. Navarro

**Affiliations:** ^1^Department of Health Promotion and Disease Prevention, Robert Stempel College of Public Health and Social Work, Florida International University, Miami, FL, USA; ^2^Opportunities Industrialization Centers of South Florida, Fort Lauderdale, FL, USA

**Keywords:** migrant workers, length of stay, risky sexual behavior, behavioral intention, alcohol use

## Abstract

The HIV rate among U.S. migrant workers is 10 times that of the national rate. The highly unstable lifestyle of migrant workers places them at heightened vulnerability to sexually transmitted infections; hence, there is a need to investigate the attitudes and sexual risk factors that may play a protective role in the transmission of HIV in this population. This study examines the association between attitudes and HIV risk behaviors among Hispanic male and female migrant workers (*n* = 255) and their length of stay (shorter length of stay as a protective factor) in Immokalee, FL, USA. Pearson’s correlation and regression analyses were utilized to analyze the relationship between HIV risk behaviors (intention to use condoms and alcohol use) with length of stay in Immokalee. Longer length of stay positively correlated with number of drinks (*p* < 0.05) and frequency of drinks (*p* < 0.01) and negatively correlated with ethnic identity search (*p* < 0.05). Regression analysis showed that length of stay predicted both behavioral intention to use condoms (*p* < 0.05) and alcohol consumption (*p* < 0.05). The findings suggest that migrant workers who are new to Immokalee may have a higher likelihood of practicing protective HIV risk behaviors and having more favorable attitudes toward risk reduction than long-timers. This study might provide important new evidence on the drivers of multiple concurrent and potential protective factors against risky sexual behaviors among Hispanic migrant workers.

## Introduction

One of the fastest growing, underserved populations in the US are migrant workers, presently exceeding over four million farmworkers (1). The majority of migrant workers are Hispanic and work in rural areas in the United States (2). This overlooked community carries a heavy burden of working in an area that involves high occupational risk as well as a number of other challenges that come along with working in the fields of the US and obstacles associated with migration, and in many cases are also undocumented (1–4). These challenges include the neighborhood they reside in, long hours, employment instability; stigma associated with migrant status, language barriers, and social isolation and place these individuals at heightened vulnerability in engaging in unhealthy coping behaviors, which increase their risk of sexually transmitted infections (STIs), especially HIV/AIDS (2). In addition to these many challenges, migrant workers have also been significantly affected by HIV/AIDS (5). The HIV seroprevalence rates reported for migrant workers are 2.6% to as high as 13.5%, 10 times the national average (6–8).

For many migrant workers, especially Hispanics, social rules and norms appear to be less restrictive in the U.S. compared to their home countries (9). This perception, coupled with the stress that often accompanies working and functioning in a different culture, may encourage the use of high risk coping mechanisms such as heavy drinking and/or risky sexual behaviors (3, 10, 11). Even when the norm involves consumption of large amounts of alcohol in their home countries, studies have shown that alcohol consumption increases with every year migrant workers reside in the U.S. (9, 12). Attitudes toward alcohol have also been found to reflect traditional gender roles (13). In a study among Latino-migrant workers in Beaufort, South Carolina, respondents indicated that drinking was not only widely accepted for men but encouraged as an act of proven masculinity and women who drank at all were not well accepted (9). Among Hispanics, males tend to consume higher levels of alcohol than their female counterparts (14). Traditional beliefs may contribute to high risk sexual behaviors among Hispanics (15). In Hispanic culture, sexual issues are highly personal and are not usually discussed, even with their sexual partners. In addition, beliefs in traditional gender roles lead to different standards for men and women. Such beliefs assume that men are more sexually active than women and that it is acceptable for men to have sexual relationships outside the purview of marriage. The gender role beliefs also tolerate sexual coercion (16).

Alcohol consumption, which is associated with uninhibited behaviors and impaired judgment, has been implicated as a risk factor for negative sexual outcomes, such as failure to use condoms and HIV transmission (17–19). Many studies have reported solicitation by sex workers, unprotected sex with casual partners, and participation in high risk sexual activities in the context of alcohol use (13, 17, 18). Although many migrant workers engage in behaviors that increase their risk of HIV, there are several factors that serve to protect them from risk. For example, a study on Mexican migrants found that those who migrated to the U.S. at an older age were less likely to engage in substance abuse compared to those who migrated when they were younger. The study also demonstrated how the “healthy migrant” effect may not be exclusive to U.S. Hispanic populations, but may also be applied to migrant workers (16, 20).

Another study that investigated protective HIV behaviors of migrant workers was conducted in Durham, North Carolina, and Mexican-sending communities. This study illustrated that condom use during vaginal sex was higher among male migrants residing in the U.S. compared to non-migrants in Mexican-sending communities, even though male migrants reported higher frequency of other HIV-related risk behaviors (21). Mexican migrants and non-migrants from their communities of origin differed significantly in their perception of the importance of condom use depending on how they perceived their sexual encounter as safe or unsafe. Mexican male migrants were more likely to believe in using condoms when engaging in sexual encounters and believed such preventative practices were of importance compared to their non-migrant counterparts. This acceptance of the use of condoms is claimed to be because of the higher incidence of multiple partners and commercial sex worker partners among these migrant workers (21–23).

Unfortunately, the literature on HIV/AIDS-risk behaviors among migrant workers is sparse, particularly in relation to protective factors. In addition to exploring sexual risk factors, it is equally important to investigate protective factors among migrant workers. This will aid researchers in designing interventions focused on encouraging the practice of protective behavior in HIV prevention strategies. Hence, the current study explores whether migrant workers’ length of stay in Immokalee acts as a protective factor that influences their attitudes toward alcohol and sexual risk behaviors. Evidence from theoretical literature suggests that shorter length of stay could act as a protective factor influencing migrant workers’ attitudes toward alcohol and sexual risk behaviors. We based our hypothesis that shorter length of stay could act as a protective factor on the health belief model. Perceived susceptibility is perceiving that oneself is at risk of sexually transmitted infection, including HIV (24–26). Increased perceived susceptibility to HIV infection has been related to increased protective behaviors like increased condom use (24, 27). We postulate that recent migrant workers have increased perceived susceptibility, an indicator of motivation to engage in protective sexual behaviors.

## Materials and Methods

### Design

Participants from an ongoing two-group randomized study comparing the effectiveness of an enhanced/adapted cognitive behavioral program with a health promotion comparison program were used for this study. Participants in the larger study were migrant Hispanic and African American workers from Immokalee, a rural agricultural area in South Florida, who were recruited using targeted sampling. The aim of the parent study was to assess the effectiveness of two community-based interventions in reducing HIV risk behaviors and increasing health behaviors among alcohol and other drug using migrant workers (28).

The inclusion criteria for participants in this sub-study were being male or female Hispanics who spoke Spanish or English, and were age 18 years or older with a history of unprotected vaginal, anal, or oral sex and/or consumption of alcohol or other drugs in the previous 3 months. The interviews were executed in both languages, English and Spanish. These analyses were conducted among Hispanic migrant workers who participated in the baseline intake interviews (*n* = 255).

The migrant workers involved in the present study are a mix of migrant workers and seasonal workers within a 10 mile radius of Immokalee, Collier County, FL, USA. The study’s definition of migrant workers conformed to the Public Health Services Act (1944) definition. The Public Health Services Act states that migrant workers are those individuals who are employed in agricultural labor, either seasonal or migratory, and live in temporary housing. The study criteria did not limit the definition of migrant workers to those who travel 75 miles or cross county lines to work. The study also included some people who work or worked indirectly in agriculture, such as in packing houses. However, the majority of migrant workers were farm workers. The Florida International University institutional review board examined (IRB) and approved the study protocol and research procedures.

### Measures

#### Length of stay

Length of stay is the number of years the participants stayed at Immokalee, FL, USA.

#### Alcohol consumption and frequency of drinking

Alcohol use was measured by asking the participants how many standard drinks they had in the past month. Photographs of drinks and sample glass sizes were shown for verification of amount of alcohol intake. Frequency of drinking is the number of drinking days in the past month.

#### Behavioral intentions

Behavioral intentions to use condoms was measured using a 15-item Behavioral Intentions Scale which focused on taking future actions to reduce the risk of transmitting HIV (e.g., “I will use a condom the next time I have sex”). This scale was derived by Klinkenberg (personal communication, 1998) by simplifying a measure used by Otto-Salaj et al. (29) and by adding an item about drinking (i.e., “I will use a condom the next time I have sex even if I’ve been drinking”). Items were scored on a 5-point scale ranging from 1 (definitely will not do) to 4 (definitely will do) and 77 (do not know). The responses were recoded with “Do not know” as mid-point. Cronbach’s alpha for the Behavioral Intention Scale was 0.90.

#### Short inventory of problems

A brief version of the short inventory of problems (SIP) was used for assessing negative consequences associated with the effects of alcohol and other drug use (e.g., “You have failed to do what is expected of you because of your drinking or drug use.”). This measure is also known as the alcohol problems measure. This questionnaire is composed of a 9-item, modified version of the drinker inventory of consequences (30, 31). Items were scored on a Likert type scale with five responses for each item, from 0 (Never/Not at all) to 4 (Daily or almost daily/Very much), yielding a range of scores from 0 to 36. The scale showed high reliability with a Cronbach’s alpha of 0.93.

#### Ethnic identity

The multigroup ethnic identity measure (MEIM) was used to assess the ethnic identity level of the participants (32). MEIM is a 12-item questionnaire, which measures participants’ level of comfort and attachment with their ethnic culture as well as members of their ethnic group. The MEIM focuses on analyzing areas of one’s feelings of commitment and/or belonging to his/her ethnic group. The MEIM score also indicates the participant’s comfort level with individuals from his or her own ethnic group (32, 33). The scores for each item use a Likert scale, ranging from 1 (strongly disagree) to 4 (strongly agree). A mean score was calculated to assess the level of ethnic identity search (first seven items) and commitment (last five items) of each participant. The higher the mean ethnic identity search score, the higher the developmental and cognitive component. The same was the case with ethnic identity commitment, the higher the commitment score, the higher the subject’s emotional attachment to their ethnic group. The Cronbach’s alpha of the ethnic identity search and commitment scales were 0.79 and 0.82, respectively.

Table 1 shows the definitions and parameters of all variables used in the study.

**Table 1:** **Variables used in the study**.

Variable name	Definition/measurement	Parameters
Length of stay	Number of years the participants stayed at Immokalee, FL, USA.	Continuous variable
Alcohol consumption	Number of standard drinks in the past month.	Continuous variable
Frequency of drinking	Number of drinking days in the past month.	Continuous variable
Behavioral intentions scale to use condoms (29)	Individual’s perceived or subjective intention of using condom during sexual intercourse.	Likert scale, ranging from 1 (definitely will not do) to 4 (definitely will do) and 77 (do not know)
Short Inventory of problems (SIP) (30, 31)	SIP measures physical, interpersonal, intrapersonal, social, and impulsive consequences of alcohol use.	Likert scale, ranging from 0 (never/not at all) to 4 (daily or almost daily/very much)
Multigroup ethnic identity measure (MEIM) (32)	Individual’s level of comfort and attachment with their ethnic culture as well as members of their ethnic group.	Likert scale, ranging from 1 (strongly disagree) to 4 (strongly agree)
Ethnic identity search (32)^a^	Developmental and cognitive component of MEIM	Likert scale, ranging from 1 (strongly disagree) to 4 (strongly agree)
Ethnic identity commitment (32)^a^	Affective component of MEIM	Likert scale, ranging from 1 (strongly disagree) to 4 (strongly agree)
HIV risk factors: number of unprotected vaginal sex episodes	Number of unprotected vaginal sex episodes in the past month.	Continuous variable
HIV risk factors: number of partners	Number of sexual partners in the past month.	Continuous variable

*^a^Ethnic identity search and ethnic identity commitment are sub-scales of MEIM*.

### Data analysis

IBM SPSS Statistics for Windows, Version 21.0 was used to analyze the data (Armonk, NY, USA: IBM Corp.). Initially, descriptive statistics were done to describe the sample population. This was followed by Pearson’s correlation of variables, which were used in the regression model to find the association between them. Finally, two separate multiple linear regression analyses were conducted after meeting all assumptions of linear regression. These analyses were conducted to predict behavioral intentions to use condoms and alcohol consumption rate. No evidence of mutlicollinearity was found among the predictor variables.

## Results

### Participants

The data were collected at baseline, prior to participation in the intervention. The sample was composed of 255 Hispanic participants (Table 2). The mean age of the sample was around 38 years with a mean educational level of 7.25 years. Average length of stay of participants was around 14 years. This included people who were staying for less than a month to up to 55 years. Nearly, 68% of participants were seasonal migrant workers. The sample was primarily male (*n* = 201, 78.8%) and majority were single (*n* = 195, 76.5%). Most of them spoke Spanish and were born in Mexico, Puerto Rico, and Cuba. Missing data was very minimal throughout the data set utilized for this study, not exceeding a range of 0–2%. A mean replacement method was used to replace missing data.

**Table 2:** **Demographic characteristics of the sample (*n* = 255)**.

Variables	Values
Age (*M* ± SD)	38.46 ± 11.59
Education (*M* ± SD)	7.25 ± 3.35
Gender *n* (%)
Male	201 (78.8%)
Female	54 (21.2%)
Marital status *n* (%)
Single	195 (76.5%)
Married	60 (23.5%)
Language *n* (%)
English	52 (20.4%)
Spanish	202 (79.2%)
Country of birth *n* (%)
USA	154 (60.4%)
Others	101 (39.6%)

### HIV risk factors

The association between HIV risk behaviors and length of stay in this sample are illustrated in Table 3. The most frequent sexual risk behavior for this sample was having vaginal sex without a condom and multiple partners. Although there was a positive correlation between longer length of stay and number of unprotected vaginal sex episodes and number of partners, the association was not significant. However, there was a significant positive correlation between number of drinks (*r* = 0.31, *p* = 0.028) and frequency of drinks (*r* = 0.28, *p* = 0.006). In addition, length of stay was inversely associated with ethnic identity search (*r* = −0.32, *p* = 0.007). Ethnic identity search was inversely correlated with number of unprotected vaginal sex episodes and number of partners. Behavioral intention to use condoms was negatively correlated with number of unprotected vaginal sex episodes (*r* = −0.09, *p* = 0.032).

**Table 3:** **Correlations among variables in the regression model (*n* = 255)**.

Variables	1	2	3	4	5	6	7	8	9
Length of stay	–	–	–	–	–	–	–	–	–
Number of unprotected vaginal sex episodes	0.31	–	–	–	–	–	–	–	–
Number of partners	0.38	0.14	–	–	–	–	–	–	–
Number of drinks	0.31*	0.06*	0.13	–	–	–	–	–	–
Frequency of drinking	0.28**	0.21	0.15	0.25	–	–	–	–	–
SIP	−0.21	0.11	0.09	0.09	0.14*	–	–	–	–
Behavioral intention	−0.08	−0.09*	−0.30	0.12	0.24	0.07	–	–	–
EI search	−0.32*	−0.20*	−0.21*	0.18	0.20	0.14	0.24	–	–
EI commitment	−0.17	0.31*	0.02	0.06	0.08	0.17	0.12	0.28	–

*p < 0.05, **p < 0.01.

*EI, ethnic identity; SIP, short inventory of problems (alcohol problems); number of drinks, number of standard drinks in the past month; frequency of drinking, number of drinking days in the past month; behavioral intention, behavioral intention to use condoms*.

*All Sexual acts and consumption of alcohol refer to the last 30 days*.

### Predicting HIV risk

The independent variables in the model predicting behavioral intentions to use condoms (Table 4) were demographic variables (age, gender, length of stay, marital status, and education), amount of alcohol consumed in last 30 days, ethnic identity subscales (ethnic identity commitment and ethnic identity explore) and SIP. The independent variables in the model predicting alcohol consumption (Table 5) were demographic variables (age, gender, length of stay, marital status, and education), ethnic identity commitment, ethnic identity explore, and SIP. In the model predicting behavioral intention, gender (β = −0.021, *p* = 0.032), length of stay (β = −0.024, *p* = 0.042), ethnic identity search (β = 0.023, *p* = 0.007), and SIP (β = 0.137, *p* = 0.012) significantly predicted behavioral intention to use condoms. In the second regression model, gender (β = −32.928, *p* = 0.026), length of stay (β = −25.856, *p* = 0.035), and SIP (β = 3.575, *p* = 0.016) significantly predicted alcohol consumed in last 30 days.

**Table 4:** **Multiple linear regression predicting behavioral intentions to use condoms (*n* = 255)**.

Independent variables	Beta estimate	Standard error	*p*-value
Age	0.014	0.003	0.678
Gender*	−0.020	0.014	0.031
Length of stay*	−0.023	0.014	0.041
Marital status	−0.017	0.007	0.318
Education	0.015	0.013	0.628
Alcohol consumption^a^	3.926	0.009	0.355
Number of partners	−1.025	0.016	0.059
EI search**^a^	0.026	0.016	0.007
EI commitment^a^	−0.031	0.021	0.269
SIP*	0.133	0.008	0.012

*p < 0.05, ** *p* < 0.01.

*^a^Transformed value*.

*EI, ethnic identity; SIP, short inventory of problems; alcohol consumption, number of standard drinks in the past month*.

*All Sexual acts and consumption of alcohol refer to the last 30 days*.

**Table 5:** **Multiple linear regression predicting alcohol consumption^a^ (*n* = 255)**.

Independent variables	Beta estimate	Standard error	*p*-value
Age	−0.263	0.272	0.529
Gender*	−32.928	12.846	0.026
Length of stay*	25.856	8.456	0.035
Marital status	9.464	5.367	0.735
Education	−2.464	1.935	0.332
EI search^a^	−12.648	9.354	0.246
EI commitment^a^	14.464	8.457	0.273
SIP*	3.575	1.010	0.016

*p < 0.05.

*^a^Transformed value*.

*EI, ethnic identity; SIP, short inventory of problems*.

*All sexual acts and consumption of alcohol refer to the last 30 days*.

## Discussion

This study was focused on exploring whether length of stay is protective in HIV risk behaviors. Several important findings in this Hispanic migrant worker study merit discussion. Hispanic migrant workers who lived in Immokalee for less time demonstrated higher intentions to use condoms and consumed less alcohol than their long-timer peers which may be a reflection of how they perceived their environment.

Condom use was inversely associated with length of stay, indicating that condom use was common among the more recent migrant workers or newcomers. Condom use was also more common in males when the full sample was examined. This finding is consistent with a previous study which revealed that newcomers were more likely to use condoms and get screened for HIV than long-timers (22). The number of sexual partners was also inversely correlated with length of stay. The relationship of length of stay and condom use and number of sexual partners might be due to their perception of casual sexual encounters in the U.S. as being risky. This study illustrates that more recent migrants tend to perceive their new environment as more risky and acknowledge that the casual sexual episodes which they pursue are risky as well. For instance, a study by Organista and Ehrlich (34) reported that migrant workers who had partners back in Mexico were about four times more likely to use condoms with casual female sexual partners than males who have their wives or partners accompanying them. Condom use variation by gender is in line with findings reported by several other study results that male migrants were more likely to believe in using condoms when engaging in sexual encounters and believed such preventative practices were of importance compared to their non-migrant counterparts (12, 21).

Predictors of the number of drinks in the last 30 days were gender, length of stay, and alcohol problems measure (sexual inventory of problems). Males were more likely to consume more alcohol than their female counterparts. Gender variation in alcohol use was reported by other studies as well (14, 35, 36). Length of stay was a significant predictor of alcohol consumption. Shorter length of stay was directly associated with fewer drinking episodes, as well as fewer drinks in the last 30 days. Previous studies reported newcomer frequency of drinking episodes to be less than migrant workers who have been in the U.S. longer (13). Although actual frequency of drinking episodes was not very high, the number of drinks per episode was, suggesting heavy or binge drinking. Alcohol consumption has been reported to increase with every year a migrant worker is in the U.S., which was evident in this study. Studies suggest that migrant workers who are not recent arrivals to the U.S. tend to be at high risk for alcohol abuse and have been found to consume high levels of alcohol to cope with their stress of working and functioning in a different culture (13, 37).

The alcohol problems measure was a significant predictor for both alcohol consumption and intentions to use condoms. Higher alcohol problems were associated with higher alcohol consumption as well as lower intentions to use condoms. Previous studies have shown negative sexual outcomes under the influence of alcohol among migrant workers (18, 34). Alcohol consumption, in many cases, may lead to risky sexual behaviors, ultimately placing the participant at higher risk for HIV. While our findings correspond with past studies on sexual risk behaviors and alcohol use, this study makes significant contributions to the existing literature on the protective role of length of stay of migrant workers in the U.S. Our findings assert the association of newcomer status with high number of condom use and lesser alcohol intake. We further add to the existing literature the important finding that length of stay could predict intention to use condom and alcohol consumption. This finding might be due to the unique perception of newcomers about the new environmental and vulnerability factors to which they are exposed.

Ethnic identity was one of the contributing factors that influenced intentions to use condoms. Previous research has demonstrated that individuals with a stronger connection with their ethnic group (ethnic identity) served as a protective factor with risky sexual behaviors and risky sexual attitudes and beliefs of individuals (38, 39). This study demonstrated that those migrant workers who were more interested in learning about their ethnic origin also practiced protective HIV risk behavior and had a more positive attitude to engaging in such protective behaviors.

Studies have shown that, although traditional beliefs may lead to high risk sexual behaviors among Hispanics, some of these beliefs may change with time due to acculturation (40). This is contrary to the findings of this study. This could be due to the fact that more than half of the participants were born in the U.S. However, more studies are needed to explore how migrant workers accommodate both mainstream and heritage culture.

There are some limitations to this study. Since this is a report on the cross-sectional portion of the study, it limits the ability to draw conclusions or make predictions of causal relationships. The sample size was also relatively small. The data reported is self-reported, which may increase recall bias. Finally, previous studies have measured drinking by number of drinks per drinking episode, which was not available in this study. This lack of information may limit the ability to draw comparisons with previous studies.

## Conclusion

Notwithstanding the methodological limitations, the findings of this study translate into relevant conclusions that newcomer status may be a protective factor against HIV risk behaviors, including condom use and alcohol consumption. As one of the few studies to date to explore shorter length of stay as a protective factor, this research provides important new evidence on the drivers of multiple concurrent and potential protective factors. These findings have important implications for health professionals who work to prevent substance abuse, sexual risk, and other HIV risk behaviors among migrant workers.

This study indicates that risk prevention interventions targeting condom use and alcohol use should be focused more on long-timers to provide them with the skills to support and enhance levels of positive change. A successful approach should meet the needs of individual migrant worker by assessing differences in problem behaviors by gender. Males, especially, need to be targeted in future HIV prevention programs since previous studies, as well as this study suggest that Hispanic males tend to be at highest risk for alcohol abuse. Future research should take into account the length of time migrant workers are in the community and include comparisons of newcomers and those who have been in the community for a longer period of time to better understand the effect of migration on HIV risk behaviors. In addition, investigations should explore if and how behavior of newcomers influences protective performance, or if unobserved factors might be implicated in this phenomenon.

## Author Contributions

H. Virginia McCoy and Nancy Shehadeh designed and conceptualized the study. Nancy Shehadeh and Muni Rubens collected the data and performed data analyses. H. Virginia McCoy, Nancy Shehadeh, Muni Rubens, and Christi M. Navarro wrote portions of the manuscript.

## Conflict of Interest Statement

The authors declare that the research was conducted in the absence of any commercial or financial relationships that could be construed as a potential conflict of interest.
